# Beijing Roundtable Addresses Research Integrity's Role in Advancing Open Science

**DOI:** 10.1002/hcs2.70089

**Published:** 2026-08-03

**Authors:** Wenqiong Lai, You Wu, Yu Sun

**Affiliations:** ^1^ International Cooperation Department Tsinghua University Press Ltd. Beijing China; ^2^ School of Healthcare Management, Tsinghua Medicine Tsinghua University Beijing China; ^3^ Associate General Editor's Office Tsinghua University Press Ltd. Beijing China

**Keywords:** artificial intelligence, medical publishing, open science, research integrity

**Abbreviations**
AI: artificial intelligence
STM: International Association of Scientific, Technical & Medical Publishers

On 17 June, a roundtable themed “Upholding Research Integrity to Advance Open Science” was convened at the 32^nd^ Beijing International Book Fair, bringing together experts from China and abroad. Six representatives from the International Association of Scientific, Technical & Medical Publishers (STM), Taylor & Francis, Tsinghua University, and Tsinghua University Press explored the emerging challenges that academic publishers and researchers face in the open science era, and proposed potential steps to strengthen research integrity.

The panel included Caroline Sutton, Chief Executive Officer of STM; Hylke Koers, Chief Information Officer, STM Solutions; Joris van Rossum, Program & Product Director, STM Solutions; Katie Peace, Vice President of Academic Partnerships, Asia, at Taylor & Francis; Xin Zhao, President of Tsinghua University Press; and You Wu, Associate Professor at the School of Healthcare Management, Tsinghua University.

Before the roundtable, Dr. Hylke Koers, Chief Information Officer of STM, introduced *STM Trends 2030* [1]. The report used space exploration as a metaphor for the pursuit of knowledge. He elaborated on the emerging trends poised to reshape scholarly communication over the next 5 years, identifying four major “solar flares”: geopolitical realignment, challenges to scientific authority, a funding shift from government to corporate research and development, and the artificial intelligence (AI)‐driven transformation of knowledge production and dissemination. He also sounded a warning about “meteor” threats that demand attention—paper mills, predatory publishing, and mounting operational costs. Amid such turbulence, Dr. Koers urged publishers to center their efforts on two priorities: building knowledge infrastructure that enables human‐machine collaboration, and fortifying authentication and trust mechanisms that safeguard research integrity.

The discussion that followed focused on four connected themes.

## 1 From Detecting Misconduct to Verifying Authenticity

The experts explored methods, tools, and theoretical perspectives for promoting research integrity. AI has made data fabrication, image manipulation, and false manuscript production easier and cheaper. In some cases, an editor may receive a complete manuscript without being able to confirm whether the authors, images, or data are genuine. The panel therefore argued that the research community should not rely only on detecting misconduct after it has occurred. Greater attention may be given to verifying authenticity from the beginning of the research process [2].

To counter this, Dr. Van Rossum advocated for the adoption of technical safeguards—including image watermarks and researcher identity verification—to enable editors to verify the authenticity of images and the institutional affiliation of authors. He further emphasized the importance of enhanced collaboration across publishers, libraries, research institutions, funders, and instrument manufacturers to enable the seamless sharing of trust signals throughout the scholarly ecosystem.

Dr. Koers made a similar point. In a world where fabricated content is cheap and widely available, it may become easier to verify that something is real than to detect whether it is fake. This requires systems that preserve information about the origin, authorship, and production of research outputs.

Publishers were advised to introduce some practical checks. Dr. Wu suggested that journals pilot asking authors to submit editable original files, vector graphics, or the code used to generate figures—researchers in good faith would have no reason to refuse, while bad actors would struggle to comply.

Some journals may not need to request all source materials at the time of submission. Katie Peace noted that some journals have adopted a “data on request” approach, which is enough to deter bad actors, while incentives such as open science badges are also playing a positive role.

## 2 Integrating Technology and Human Judgment

The speakers agreed that technology alone is not enough to preserve research integrity. Automated systems can be used to help identify unusual patterns, suspicious citations, manipulated images, or possible paper mill activity [3]. However, these signals still need to be interpreted by experienced editors, reviewers, and researchers. Dr. Caroline Sutton likened it to a system of checks and balances, in which publisher systems flag anomalies and humans verify the systems, with the two acting as a check on each other.

Dr. Sutton suggested that publishers could join the STM Integrity Hub, using its screening tools to perform a baseline review of submissions—a foundational safeguard that larger publishers can further strengthen by layering on their own proprietary or third‐party technologies. Dr. Wu emphasized the value of involving more active researchers in the publishing process. Researchers who use generative AI tools in their own work may be more familiar with the appearance and limitations of AI‐generated text and figures. They may recognize direct AI outputs, unusual visual styles, fabricated labels, or text that has been generated rather than simply polished.

In practice, however, this places additional demands on peer reviewers and publishing teams [4]. Therefore, publishers were advised to place screening tools earlier in the process. These tools would be able to provide useful information before a manuscript reaches an academic editor.

Ms. Peace added that publishers, too, have a role to play in educating and supporting authors, reviewers, and editors, helping them better understand the challenges we now face and the standards and expectations that apply. Dr. Van Rossum stressed that openness also calls for deeper collaboration among publishers, who are urged to share candidly with one another the patterns of misconduct they detect and the practical lessons they have learned in tackling paper mills.

## 3 Turning Policies Into Workable Practices

Many publishers and journals already have policies on data sharing, image manipulation, authorship, conflicts of interest, and the use of AI. Ms. Peace noted that policies themselves are updated frequently, but the key lies in implementation—they need to be supported by standard operating procedures and ongoing training so that editors and reviewers truly understand and can apply the policies.

The panel stressed that research integrity should be built into routine workflows. Submission systems can ask for ethics approval, author contributions, conflict‐of‐interest statements, data availability, and original research files. Screening tools may be used to check references, images, identities, and submission patterns. Clear procedures are also needed when a concern is identified.

Dr. Sutton noted that human involvement means that some problems will still be missed. Editors and reviewers may make mistakes. In rare cases, individuals may also fail in their gatekeeping responsibilities. Retractions therefore remain an important corrective mechanism. They allow the scholarly record to be updated when serious problems are discovered after publication.

## 4 Research Integrity as a Shared and Upstream Responsibility

Many integrity problems begin long before a paper reaches a journal. They may arise during study design, data collection, analysis, authorship decisions, or institutional evaluation. Publishers cannot address these problems alone. The panel called for closer cooperation among publishers, researchers, universities, funders, libraries, technology providers, and research instrument manufacturers.

Dr. Sutton proposed that stakeholders clearly define the respective roles of funders, research institutions, and publishers. She also expressed the hope that every institution and funding body would appoint a dedicated research integrity officer—someone well versed in local regulations and readily accessible for coordination.

Xin Zhao urged Chinese university presses to join multilateral frameworks on research integrity governance. He noted that the Hong Kong Principles, adopted at the World Conference on Research Integrity held in China, serve as a testament to the country's growing influence in this domain [5]. He also suggested that publishers draw on platforms such as Committee on Publication Ethics and Retraction Watch to detect misconduct, and that consensus principles be translated into standardized tools. Regarding legal compliance, he emphasized that data handling practices may need to take into account both *Data Security Law of the People's Republic of China* [6] and the European Union's *General Data Protection Regulation* [7], and suggested that collaboration with legal experts—potentially under the guidance of the Committee on Publication Ethics—could be a useful approach to developing clearer data‐sharing standards.

The panel also discussed the pressure created by current research evaluation systems. A strong focus on publication numbers, high‐impact journals, and positive findings can increase the risk of questionable practices [8]. Dr. Wu argued that recognition also be given to the scientific value of null or negative findings, so as to remove the incentive for researchers to manipulate data in pursuit of positive results. A well‐designed study remains valuable even when it does not produce a statistically significant result. Dr. Sutton observed that while international discussions on research assessment reform have made some headway, overall progress remains slow. Dr. Koers and Ms. Peace also highlighted the need for better incentives. Open data, replication studies, reusable code, and other contributions should be recognized in research assessment. Ms. Peace added that Taylor & Francis has created a dedicated Vice President of Impact position, tasked with exploring multidimensional metrics for evaluating research quality.

During the closing reflections, the speakers chose several words to summarize the discussion: “thoughtfulness,” “trust,” “collaboration,” “open,” and the Chinese concept of *liangzhi*—encompassing both “good knowledge” and “conscience”—to express the scholarly community's aspiration to uphold quality and integrity in their work. Despite significant changes in research policies and the rapid evolution of AI technologies, all participants expressed a commitment to addressing these uncertainties through an open and collaborative system in which researchers, institutions, funders, and publishers share responsibility for maintaining trust in science.

## Author Contributions

**Wenqiong Lai:** conceptualization (equal), writing–review and editing (equal). **You Wu:** writing–original draft (equal). **Yu Sun:** conceptualization (equal), resources (lead).

## Funding

The authors have nothing to report.

## Ethics Statement

The authors have nothing to report.

## Consent

The authors have nothing to report.

## Conflicts of Interest

Dr. Yu Sun is the Director of the *Health Care Science* Editorial Office. To minimize bias, she was excluded from all editorial decision making related to the acceptance of this article for publication. Wenqiong Lai is the director of International Cooperation Department at Tsinghua University Press Ltd. The remaining author declares no conflict of interest.

## Data Availability

Data sharing is not applicable to this article as no new data were created or analyzed in this article.

## References

[1] “STM Trends: A Futurecast,” STM, accessed June 24, 2026, https://stm-assoc.org/what-we-do/strategic-areas/standards-technology/stm-trends-2030/.

[2] M. Naddaf , “How to Spot Fake Scientists and Stop Them From Publishing Papers,” Nature 646, no. 8086 (2025): 792–794, 10.1038/d41586-025-03341-9. 41125782

[3] Y. Wu and H. Wang , “Redefining Medical Publishing in the Artificial Intelligence Era,” Health Care Science 4, no. 4 (2025): 314–315, 10.1002/hcs2.70026. 40861516 PMC12371719

[4] F. Beigel , “The Transformative Relation Between Publishers and Editors: Research Quality and Academic Autonomy at Stake,” Quantitative Science Studies 6 (2025): 154–170, 10.1162/qss_a_00343.

[5] “Hong Kong Principles,” World Conferences on Research Integrity, accessed July 1, 2026, https://www.wcrif.org/guidance/hong-kong-principles.

[6] “Data Security Law of the People's Republic of China,” The Supreme People's Procuratorate of the People's Republic of China, accessed July 1, 2026, https://en.spp.gov.cn/2021-06/10/c_948426.htm.

[7] “General Data Protection Regulation (GDPR),” EUR‐lex, accessed July 1, 2026, https://eur-lex.europa.eu/EN/legal-content/summary/general-data-protection-regulation-gdpr.html.

[8] X. Hu , “Constructing an Effective Evaluation System to Identify Doctors' Research Capabilities,” Health Care Science 3, no. 1 (2024): 67–72, 10.1002/hcs2.82. 38939166 PMC11080928

